# Programming of Adiposity in Childhood and Adolescence: Associations With Birth Weight and Cord Blood Adipokines

**DOI:** 10.1210/jc.2016-2342

**Published:** 2016-11-14

**Authors:** Joy Simpson, Andrew D. A. C. Smith, Abigail Fraser, Naveed Sattar, Robert S. Lindsay, Susan M. Ring, Kate Tilling, George Davey Smith, Debbie A. Lawlor, Scott M. Nelson

**Affiliations:** 1 School of Medicine, University of Glasgow, Glasgow G12 8QQ, United Kingdom;; 2 Medical Research Council Integrative Epidemiology Unit, University of Bristol, Bristol BS8 2BN, United Kingdom;; 3 School of Social and Community Medicine, University of Bristol, Bristol BS8 1TH, United Kingdom; and; 4 Institute of Cardiovascular and Metabolic Medicine, British Heart Foundation Glasgow Cardiovascular Research Centre, University of Glasgow, Glasgow G12 8TA, United Kingdom

## Abstract

**Context:**

Exposure to maternal adiposity during pregnancy is associated with higher offspring birth weight and greater adiposity through childhood and adult life. As birth weight reflects the summation of lean and fat mass, the extent to which fat mass at birth tracks into later life is unknown.

**Objective:**

To determine whether fat mass at birth is associated with child and adolescent adiposity.

**Design, Setting, and Participants:**

UK birth cohort with markers of neonatal fat mass; cord blood leptin, adiponectin, and birth weight and adiposity outcomes at age 9 (n = 2775) and 17 years (n = 2138).

**Main Outcomes:**

Offspring body mass index (BMI), waist circumference, dual-energy X-ray absorptiometry–determined fat mass, and obesity at age 9 and 17 years.

**Results:**

Higher cord blood leptin was associated with higher *z* scores of fat mass [difference in mean per 10 pg/mL: 0.03 standard deviation (SD); 95% confidence interval (CI), 0.00 to 0.06], waist circumference (0.04 SD; 95% CI, 0.00 to 0.07), and BMI (0.04 SD; 95% CI, 0.00 to 0.08) at age 9. However, by age 17 the adjusted results were attenuated to the null. Cord blood adiponectin was not associated with measures of adiposity at age 9. At age 17, cord blood adiponectin was positively associated with fat mass (0.02 SD per 10 μg/mL; 95% CI, 0.02 to 0.03) and waist circumference (0.04 SD per 10 μg/mL; 95% CI, 0.03 to 0.05). Birth weight was positively associated with waist circumference (0.03 SD per 100 g; 95% CI, 0.02 to 0.04) and BMI (0.02 SD per 100 g; 95% CI, 0.00 to 0.03), but not fat mass or odds of obesity. Cord blood leptin and adiponectin were not associated with obesity at either age.

**Conclusions:**

Increased cord blood leptin and adiponectin, known surrogates of fetal fat mass, were weakly associated with increased fat mass in late childhood and adolescence, respectively.

Exposure to maternal adiposity during pregnancy is associated with higher offspring birth weight and greater adiposity through childhood and adult life (1). Developmental overnutrition has been proposed as a mechanism by which excessive transplacental passage of nutrients facilitates the development of larger babies with greater fat mass. Evidence from within-sibling studies, comparisons of maternal and paternal exposures, and the use of genetic variants as proxies for the maternal exposures support maternal adiposity and developmental overnutrition causing greater adiposity in offspring at birth (2–4). However, whether this causal effect extends to long-term offspring adiposity is unclear. A longer term effect may occur as a result of tracking of birth fatness across the life course. However, because birth weight is unable to distinguish relative contributions of lean vs fat mass (5, 6), few studies to date have been able to determine the extent to which greater fat mass at birth tracks into later life.

Umbilical cord blood leptin is widely recognized as an accurate biomarker for neonatal fat mass (7). Maternal exposures, including maternal adiposity, which may cause developmental overnutrition, have been associated with increased cord leptin and neonatal adiposity at birth (8, 9). In animal models, fetal leptin has also been proposed to contribute the long-term programming of hypothalamic feeding circuits, thereby providing a means by which leptin can influence long-term adiposity independent of tracking of adiposity from birth (10). However, use of cord blood leptin in determining whether neonatal fat mass tracks across childhood has been limited (11–14). This primarily reflects the scarcity of large prospective birth cohorts with cord blood samples and detailed measures of offspring adiposity as well as potential confounders. Studies that have made some assessment of this to date have had relatively small sample sizes (N = 56 to 588) (11–14), and we are not aware of any study having followed children beyond age 7 years. These studies have reported inconsistent results, with higher cord leptin associated with both a lower (11) and higher (12) body mass index (BMI) at age 3 years, and a higher BMI at age 7 years (14).

Neonatal levels of adiponectin, which has insulin-sensitizing effects in adults, are approximately 4 to 7 times higher than maternal levels. Furthermore, whereas maternal circulating concentrations of adiponectin are inversely associated with BMI, higher levels of cord blood adiponectin are associated with higher birth weight (11, 15). That higher cord blood adiponectin concentrations might reflect increased fat mass in neonates is suggested by mouse studies where overexpression of fetal adiponectin was positively related to the size of fat depots in early life, whereas adiponectin knockout fetuses display lower body weight and lower fat content (16). Given this effect of adiponectin on body composition, specifically its fat deposition–enhancing effect in mice, and the known relationships of leptin in humans to fat mass, we hypothesized that both cord blood leptin and adiponectin would be positively associated with offspring adiposity in prepubertal children and adolescents.

The aim of this study was to determine whether cord blood leptin and adiponectin were positively associated with later obesity, BMI, waist circumference, and fat mass and whether this is independent of maternal BMI. For comparison, we also examined associations of birth weight with these outcomes.

## Research Design and Methods

### Study population

**Figure 1.**
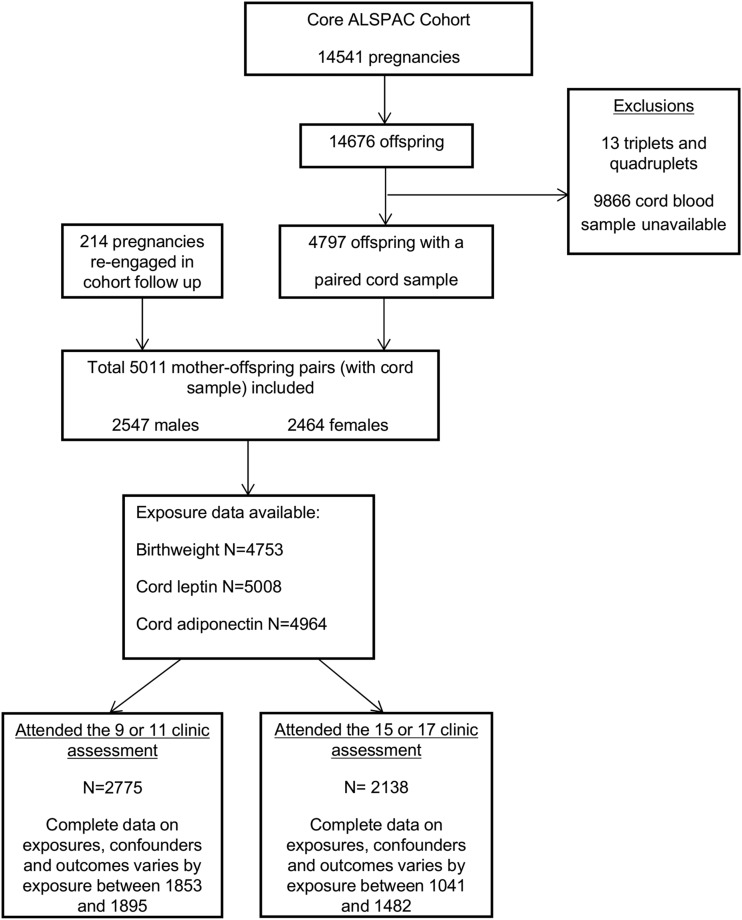
ALSPAC participant flowchart.

The Avon Longitudinal Study of Parents and Children (ALSPAC) is a prospective birth cohort study investigating the health and development of children (17, 18). The study Web site contains details of all of the data that are available through a fully searchable data dictionary (http://www.bris.ac.uk/alspac/researchers/data-access/data-dictionary/). Ethical approval was obtained from the ALSPAC Law and Ethics Committee and the National Health Service local ethics committees. A total of 14,541 women were initially enrolled, with 5011 mother/offspring pairs having a suitable cord blood sample. A detailed outline of the exclusion criteria for the analysis reported in this study and numbers with missing data are shown in Fig. 1. We included participants when they had (1) attended and completed assessments at either the 9- or 11-year clinic assessment, or (2) attended the 15- or 17-year clinic assessment. The eligible cohort for the current analysis was 2775 mother/offspring pairs at age 9 to 11 years and 2138 mother/offspring pairs at age 15 to 17 years.

### Cord blood assays

Cord blood samples were collected at the time of delivery, initially stored at 4°C for 0 to 8 days before plasma was separated and then stored at −20°C before being transferred to long-term storage at −80°C. Cord blood leptin and adiponectin were measured using commercially available enzyme-linked immunosorbent assay kits [Quantikine human leptin immunoassay (catalog no. PDLP00), Quantikine human total adiponectin/Acrp30 (catalog no. PDRP300), both R&D Systems, Minneapolis, MN]. Analysis of the cord blood was completed within a maximum of 3 freeze-thaw cycles and remained at −80°C in between thaws. The interassay coefficients of variability were 9.5% for leptin and 3.2% for adiponectin.

### Obstetric/perinatal data

Six trained research midwives retrospectively extracted data from obstetric medical records, and error rates were consistently <1%. These data included weight at every antenatal clinic visit (used to determine gestational weight gain), complications during pregnancy (hypertensive or diabetic disorders), and mode of delivery. Gestational age and sex and birth weight of offspring were obtained from hospital records at the time of birth. Maternal age, prepregnancy height and weight, smoking status (defined as never smoked, smoked before but not during pregnancy, and smoked during pregnancy), parity, occupational social class, and highest educational attainment were obtained from questionnaires completed by the mothers in early and advanced stages of pregnancy. Occupation was used to allocate social class groups using the 1991 British Office of Population and Census Statistics classification.

### Offspring childhood and adolescent adiposity measurements

Identical protocols were used at all follow-up clinics. At each clinic assessment, participants’ age in months was recorded and their weight and height were measured in light clothing and without shoes. Weight was measured to the nearest 0.1 kg using Tanita scales. Height was measured to the nearest 0.1 cm using a Harpenden stadiometer. Dual-energy X-ray absorptiometry scans were used to measure total fat mass. Waist circumference was measured to the nearest 1 mm at the midpoint between the lower ribs and the pelvic bone with a flexible tape and with the child breathing normally. Offspring obesity was classified using BMI and criteria defined by the International Obesity Task Force (19).

### Statistical analysis

The relationship between exposures (birth weight and cord blood adipokines) and outcomes (BMI, waist circumference, and fat mass at ages 9 to 11 years and 15 to 17 years) was examined by Spearman correlation. Linear (offspring BMI, waist circumference, and fat mass) and logistic (offspring obesity) regression models were used to examine the associations between birth weight and cord blood measures and offspring BMI, waist circumference, fat mass, and obesity at age 9 and 17 years. Offspring waist circumference and fat mass were log transformed to produce approximately normal distributions of regression model residuals. Within-cohort logged fat mass and waist circumference *z* scores (participant value minus mean for the sex and age category ÷ standard deviation for the sex and age category) were created using 1 year age categories. BMI *z* scores were created using the UK 1990 British growth reference (20). Birth weight was adjusted for sex, gestational age, and number of offspring (singletons or twins) using nonlinear regression fitting a Gompertz curve.

Three incremental analyses were performed to adjust for potential confounders (Supplemental Fig. 1). The basic model (model 1) adjusted for offspring sex and age at outcome measurement alone (and offspring height when fat mass is the outcome). In model 2 we additionally adjusted for maternal confounders (age, smoking, parity, occupational social class, education, and prepregnancy BMI). In the fully adjusted model (model 3) we additionally adjusted for pregnancy characteristics (gestational age at birth, mode of delivery, gestational weight gain, hypertensive and diabetic disorders of pregnancy). In these analyses, because we have scaled the exposures (birth weight, cord blood leptin, and adiponectin) and outcomes (BMI, waist circumference, and fat mass) on their standard deviations, the resultant differences in means from the multivariable linear regression models are equivalent to partial (adjusted) correlation coefficients and can be interpreted in this way.

There were small amounts of missing data on some covariables included in the multivariable models (Fig. 1). Twenty imputation data sets were generated by chained equations (21), with all cord exposures, birth weight, the covariates specified for model 3, and the measurements from the 11-year clinic and 15-year clinic informing imputation of missing values in the 9-year clinic and 17-year clinic, respectively (hereafter referred to as 9 and 17 year). The distributions of observed and imputed variables were similar (Supplemental Table 1). In the following sections, we present results from the imputed datasets and, for comparison, present results from those with complete confounders (N = 1041 to 1776) in Supplemental Material (Supplemental Tables 5–8).

All statistical analyses were performed using Stata (version 13.0) software (StataCorp, College Station, TX).

## Results

Table 1 summarizes the maternal and offspring characteristics for those participants with cord blood measures who completed at least 1 clinic assessment, with Supplemental Table 1 demonstrating the similarity of the observed and imputed data. Supplemental Table 2 shows the Spearman correlation between exposures (birth weight and cord blood adipokines) and outcomes (markers of anthropometry at age 9 and 17 years). Birth weight was positively correlated with cord blood leptin (n = 4751, *r* = 0.33) and, to a lesser degree, with cord blood adiponectin (n = 4707, *r* = 0.14). Cord leptin and adiponectin were positively correlated (n = 4962, *r* = 0.11). Birth weight and leptin also positively correlated with fat mass, BMI, and waist circumference at age 9 and 17 years. There was a weak inverse association between cord adiponectin and waist circumference and BMI at age 9. Among those participants with assessments at both clinics (at age 9 and 17 years), measurements at each clinic were highly correlated (0.74 for BMI, 0.74 for fat mass, and 0.66 for waist circumference).

**Table 1.** **Maternal and Offspring Characteristics**

	Attended at Least 1 Clinic Assessment (n = 2955)	
	N Observations (%)	Median (IQR)
Maternal characteristics		
Age	2914	29 (26, 32)
Smoking		
Never	2103 (73.8)	
Before, not during pregnancy	212 (7.4)	
During pregnancy	533 (18.7)	
BMI	2587	22.2 (20.5, 24.4)
Parity		
0	1274 (45.5)	
1	1011 (36.1)	
2	383 (13.7)	
3	101 (3.6)	
4+	30 (1.1)	
Education		
Left school at 16	1713 (61.3)	
A level	689 (24.8)	
Degree	391 (14.0)	
Social class		
I (least disadvantaged)	140 (5.9)	
II	807 (33.7)	
IIIa	1038 (43.5)	
IIIb	162 (6.8)	
IV	203 (8.5)	
V (most disadvantaged)	40 (1.7)	
Pregnancy characteristics		
Gestational age at birth, wk	2914	40 (39, 41)
Model of delivery		
Spontaneous	2253 (77.9)	
Breech	36 (1.3)	
Cesarean section	249 (8.6)	
Forceps	167 (5.8)	
Vacuum	154 (5.3)	
Other	32 (1.1)	
Gestational weight gain, kg	2668	12.5 (9.5, 15.2)
Hypertension and pre-eclampsia		
No hypertensive disorders	2449 (84.5)	
Hypertension, no pre-eclampsia	420 (13.9)	
Hypertension and pre-eclampsia	49 (1.7)	
Diabetes		
No glycosuria or diabetes	2651 (95.8)	
Existing diabetes	10 (0.4)	
Gestational diabetes	16 (0.6)	
Glycosuria	91 (3.3)	
Offspring characteristics		
Sex		
Male	1414 (47.9)	
Female	1541 (52.2)	
Birth weight, kg	2891	3.5 (3.1, 3.8)
Cord leptin, pg/mL	2952	6.4 (3.6, 12.1)
Cord adiponectin, µg/mL	2927	75.7 (53.6, 98.4)
Height, cm	Age 9: 2561	140 (136, 144)
	Age 11: 2363	151 (146, 156)
	Age 15: 1816	169 (163, 175)
	Age 17: 1648	170(164, 178)
Fat mass, kg	Age 9: 2460	7.3 (4.9, 11.2)
	Age 11: 2327	10.0 (6.8, 15.7)
	Age 15: 1716	13.7 (8.6, 20.6)
	Age 17: 1594	16.7 (11.0, 23.5)
Waist circumference, cm	Age 9: 2574	61.1 (57.4, 66.6)
	Age 11: 2362	66.0 (61.8, 73.5)
	Age 15: 1475	75.4 (71.0, 81.5)
BMI, kg/m^2^	Age 9: 2560	17.0 (15.7, 19.1)
	Age 11: 2359	18.4(16.6, 21.0)
	Age 15: 1811	20.7 (19.0, 23.1)
	Age 17: 1647	22.0 (20.2, 24.7)
Obese	Age 9: 102 (4.0)	
	Age 11:116 (4.9)	
	Age 15: 78 (4.3)	
	Age 17:105 (6.4)	
Age at attendance, y	Age 9: 2583	9.8 (9.6, 10.0)
	Age 11: 2378	11.8 (11.6, 11.8)
	Age 15: 1838	15.4 (15.3, 15.6)
	Age 17: 1695	17.8 (17.6, 17.9)

Figures are numbers (%) unless stated otherwise.

Abbreviation: IQR, interquartile range.

Table 2 shows the multivariable associations between cord blood leptin, adiponectin, and birth weight and *z* scores of offspring fat mass, waist circumference, BMI, and obesity at age 9 years. Cord blood leptin was positively associated with fat mass, waist circumference, and BMI at age 9 (model 1). The effect size was largely attenuated with adjustment for maternal and pregnancy characteristics (Table 2), with the individual univariate association of maternal and pregnancy characteristics on cord leptin, cord adiponectin, and birth weight shown in Supplemental Table 3. A similar but weaker pattern was observed for measures at age 17 where cord leptin was associated with *z* scores of fat mass, waist circumference, and BMI and with the risk of obesity (Table 3). These associations were similarly attenuated to the null after adjustment for potential confounders.

**Table 2.** **Associations of Birth Weight and Cord Blood Analyte With Fat Mass, Waist Circumference and BMI *z* Scores, and Obesity Outcome at Age 9 Years**

Exposure	Outcome	Fat Mass *z* Score*^a^*			Waist Circumference *z* Score			BMI *z* Score			Obesity		
		Coefficient	95% CI	*P*	Coefficient	95% CI	*P*	Coefficient	95% CI	*P*	OR	95% CI	*P*
Leptin, per 10 pg/mL	Model 1	0.07	0.04, 0.10	<0.001	0.08	0.05, 0.12	<0.001	0.11	0.07, 0.15	<0.001	1.15	1.00, 1.31	0.046
	Model 2	0.04	0.00, 0.07	0.023	0.05	0.01, 0.08	0.008	0.06	0.02, 0.10	0.003	1.00	0.85, 1.17	0.993
	Model 3	0.03	0.00, 0.06	0.086	0.04	0.00, 0.07	0.045	0.04	0.00, 0.08	0.029	0.95	0.81, 1.12	0.548
Adiponectin, per 10 µg/mL	Model 1	0.00	−0.01, 0.01	0.828	−0.01	−0.02, 0.00	0.072	0.00	−0.02, 0.01	0.602	0.99	0.94, 1.05	0.845
	Model 2	0.00	−0.01, 0.01	0.916	−0.01	−0.02, 0.00	0.118	0.00	−0.01, 0.01	0.858	1.00	0.94, 1.05	0.874
	Model 3	0.00	−0.01, 0.01	0.875	−0.01	−0.02, 0.00	0.100	0.00	−0.01, 0.01	0.767	0.99	0.94, 1.05	0.834
Birth weight, per 100 g*^b^*	Model 1	0.01	0.00, 0.02	0.006	0.03	0.03, 0.04	<0.001	0.04	0.03, 0.05	<0.001	1.06	1.02, 1.10	0.006
	Model 2	0.00	0.00, 0.01	0.192	0.03	0.02, 0.04	<0.001	0.04	0.03, 0.04	<0.001	1.03	0.99, 1.07	0.193
	Model 3	0.00	−0.01, 0.01	0.741	0.02	0.02, 0.03	<0.001	0.03	0.02, 0.04	<0.001	1.01	0.96, 1.05	0.852

For this study, N= 2775. Model 1 was adjusted for offspring sex and age at measurement. Model 2 was adjusted for offspring sex, age at measurement, and maternal confounders (age, smoking, parity, occupational social class, education, and prepregnancy BMI). Model 3 was adjusted for offspring sex, age at measurement, and maternal confounders plus pregnancy confounders (gestational age at birth, mode of delivery, gestational weight gain, hypertensive disorders, and diabetic disorders of pregnancy).

Abbreviations: CI, confidence interval; OR, odds ratio.

^*a*^ Fat mass adjusted for height.

^*b*^ Birth weight adjusted for sex, gestational age, and singleton/twin pregnancy.

**Table 3.** **Associations of Birth Weight and Cord Blood Analyte With Fat Mass, Waist Circumference (at Age 15 Years), BMI *z* Scores, and Obesity Outcomes at Age 17 Years**

Exposure	Outcome	Fat Mass *z* *Score**^a^*			Waist Circumference *z* Score			BMI *z* Score			Obesity		
		Coefficient	95% CI	*P*	Coefficient	95% CI	*P*	Coefficient	95% CI	*P*	OR	95% CI	*P*
Leptin, per 10 pg/mL	Model 1	0.07	0.03, 0.11	<0.001	0.06	0.02, 0.10	0.003	0.09	0.04, 0.14	<0.001	1.13	0.99, 1.28	0.060
	Model 2	0.02	−0.02, 0.06	0.263	0.01	−0.03, 0.05	0.545	0.03	−0.02, 0.07	0.272	0.96	0.83. 1.12	0.629
	Model 3	0.02	−0.02, 0.05	0.444	0.01	−0.03, 0.05	0.598	0.02	−0.03, 0.06	0.481	0.95	0.81, 1.11	0.497
Adiponectin, per 10 µg/mL	Model 1	0.01	0.00, 0.03	0.034	0.01	0.00, 0.03	0.033	0.01	−0.01, 0.02	0.245	1.03	0.98, 1.08	0.238
	Model 2	0.02	0.00, 0.03	0.006	0.02	0.00, 0.03	0.008	0.01	0.00, 0.03	0.076	1.04	0.99, 1.10	0.660
	Model 3	0.02	0.00, 0.03	0.007	0.02	0.00, 0.03	0.008	0.01	0.00, 0.03	0.080	1.05	0.99, 1.10	0.613
Birth weight, per 100 g*^b^*	Model 1	0.02	0.02, 0.03	<0.001	0.04	0.03, 0.05	<0.001	0.04	0.03, 0.05	<0.001	1.05	1.02, 1.09	0.004
	Model 2	0.01	0.00, 0.02	0.010	0.03	0.02, 0.04	<0.001	0.02	0.01, 0.03	<0.001	1.02	0.98, 1.06	0.241
	Model 3	0.01	0.00, 0.02	0.098	0.03	0.02, 0.04	<0.001	0.02	0.01, 0.03	<0.001	1.01	0.97, 1.05	0.516

For this study, N = 2138. Model 1 was adjusted for offspring sex and age at measurement. Model 2 was adjusted for offspring sex, age at measurement, and maternal confounders (age, smoking, parity, occupational social class, education, and prepregnancy BMI). Model 3 was adjusted for offspring sex, age at measurement, and maternal confounders plus pregnancy confounders (gestational age at birth, mode of delivery, gestational weight gain, hypertensive disorders, and diabetic disorders of pregnancy).

Abbreviations: CI, confidence interval; OR, odds ratio.

^*a*^ Fat mass adjusted for height.

^*b*^ Birth weight adjusted for sex, gestational age, and singleton/twin pregnancy.

Cord blood adiponectin was not associated with any measures of adiposity at age 9 years (Table 2). At age 17 years, cord blood adiponectin was positively associated with fat mass and waist circumference, with the effect size strengthened after adjustment for maternal and pregnancy characteristics (Table 3).

Birth weight was positively associated with fat mass, waist circumference, and BMI at age 9 years and 17 years and showed a weak relationship with obesity in both age groups (Tables 2 and 3). After adjustment for maternal and pregnancy characteristics, increasing birth weight remained associated with greater waist circumference and BMI, with the association with fat mass and obesity attenuated to the null.

Results did not differ substantially when absolute measures of adiposity at age 9 years (Supplemental Table 4) or age 17 years were considered (Supplemental Table 5). Results were similar for nonimputed analyses but with wider confidence intervals (Supplemental Tables 6–9).

## Discussion

In this prospective birth cohort study, cord leptin, a marker of neonatal fat mass, exhibited relatively weak relationships with later measures of adiposity. These were largely attenuated by adjustment for maternal factors, particularly in later childhood. In contrast, adiponectin exhibited no relationship with measures of fat mass at age 9 years and showed a weak relationship with fat mass and waist circumference at age 15 years. Neither cord leptin nor adiponectin was associated with the risk of being classed as obese in late childhood or adolescence. Taken together, this would suggest that neonatal fat mass *per se* has a limited contribution in determining fat mass in adolescence.

To date, birth weight, as a proxy for intrauterine growth, and its relationship to adult BMI have been extensively studied. Similar to our findings, studies principally demonstrate a positive association between birth weight and childhood and adult fat mass, BMI, and waist circumference (22). To try to examine whether birth weight is simply acting as a surrogate for neonatal fat mass, we previously used the Ponderal index (birth weight/length^3^), a measure of fatness, and demonstrated positive associations with lean body mass, total body fat, and the fat-to-lean mass ratio at age 9 years (23). Although this suggests that neonatal fat mass is related to later adiposity, the Ponderal index is a relatively poor measure of neonatal total body fat (24).

To extend and improve on this work, the present study used cord blood leptin, a strong correlate of neonatal fat mass as assessed by skinfolds or total body electrical conductivity (25), and adiponectin, which in mouse studies is suggested to be a further positive correlate of fat mass (16). That cord blood leptin was positively associated with several adiposity measures, and specifically fat mass *z* score at age 9 years, suggests that there is either accretion of adipose tissue during intrauterine life that is maintained throughout childhood, the propensity to develop fat mass may be maintained, or there is a direct effect on the programming of hypothalamic feeding circuits. However, given our observed effect size, the contribution of neonatal fat to later fat mass is likely to be small. For example, a 10 pg/mL increase in cord leptin would be associated with a BMI increase from 22 to 22.1 kg/m^2^ at age 9 years.

In accordance with some (26–28) but not all (29, 30) previous studies, we observed that adiponectin was weakly positively correlated with birth weight and cord leptin. We found some evidence for weak associations of cord blood adiponectin with adiposity at the older age (15–17) but none that this was mediated by increased (and persistent) fat mass through childhood. Why adiponectin is not related to adiposity outcomes in earlier childhood, as leptin is, is not clear. Perhaps these associations emerge after puberty, which has a major impact on body composition and adipocyte number (31). It is also possible that given the multiple tests performed, some associations are due to chance, and we would caution against assuming these associations are real without further replication.

As previously shown in this cohort (32), we observed consistent positive associations of birth weight with later BMI and waist circumference in both early childhood and adolescence, although null associations (coefficients equal to 0) were found for fat mass at both ages.

Our study has several strengths, including its size, duration of follow-up, and the availability of data on a range of maternal, pregnancy, and social factors to facilitate a robust analysis. This is also 1 of the very few studies with dual-energy X-ray absorptiometry measurements of body composition at different time points, thereby overcoming the potential increase in overall mass attributed to the expected increase in bone density that results from increased adiposity. We do, however, acknowledge some limitations. The number of children who were overweight or obese was smaller than many contemporary populations. That birth weight and cord measures were not associated with the risk of being obese may reflect this. Another limitation of the study is the loss to follow-up. Our results may be biased if associations were substantially different among excluded participants due to conditioning on the variables in the model. We acknowledge that engaged participants may exhibit different characteristics at birth beyond gestational age and birth weight that are representative of the whole cohort, and also for the 2 outcomes. Replication of our analyses in additional birth cohorts with different metabolic risk profiles would strengthen our findings. Cord blood sample degradation may have contributed to variability, but leptin and adiponectin do appear to be stable with long-term storage (33–38). This is in stark contrast to C-peptide, the preferred index of fetal glucose exposure, which we were unable to measure accurately due to degradation with long-term storage, a phenomenon previously reported by others (39).

In conclusion, we found that cord blood leptin and adiponectin, known surrogates of fetal fat mass, were weakly positively associated with some measures of fat mass in late childhood and adolescence. That these associations were robust to a wide range of confounders that may reflect intrauterine, maternal, and shared environmental exposures suggests that neonatal fat mass may track into later life. However, we acknowledge replication of our findings in cohorts with a different risk profile is critical, and that the magnitude of the observed associations is small, potentially limiting the impact that neonatal life adiposity has on later outcomes.

## References

[1] Nelson SM, Matthews P, Poston L Maternal metabolism and obesity: modifiable determinants of pregnancy outcome. Hum Reprod Update. 2009;16:255–275. 19966268 10.1093/humupd/dmp050 PMC2849703

[2] Lawlor DA, Relton C, Sattar N, Nelson SM Maternal adiposity—a determinant of perinatal and offspring outcomes? Nat Rev Endocrinol. 2012;8:679–688. 23007319 10.1038/nrendo.2012.176

[3] Lawlor DA The Society for Social Medicine John Pemberton Lecture 2011. Developmental overnutrition—an old hypothesis with new importance? Int J Epidemiol. 2013;42:7–29. 23508404 10.1093/ije/dys209

[4] Tyrrell J, Richmond RC, Palmer TM, Feenstra B, Rangarajan J, Metrustry S, Cavadino A, Paternoster L, Armstrong LL, De Silva NM, Wood AR, Horikoshi M, Geller F, Myhre R, Bradfield JP, Kreiner-Moller E, Huikari V, Painter JN, Hottenga JJ, Allard C, Berry DJ, Bouchard L, Das S, Evans DM, Hakonarson H, Hayes MG, Heikkinen J, Hofman A, Knight B, Lind PA, McCarthy MI, McMahon G, Medland SE, Melbye M, Morris AP, Nodzenski M, Reichetzeder C, Ring SM, Sebert S, Sengpiel V, Sorensen TI, Willemsen G, de Geus EJ, Martin NG, Spector TD, Power C, Jarvelin MR, Bisgaard H, Grant SF, Nohr EA, Jaddoe VW, Jacobsson B, Murray JC, Hocher B, Hattersley AT, Scholtens DM, Davey Smith G, Hivert MF, Felix JF, Hypponen E, Lowe WL Jr, Frayling TM, Lawlor DA, Freathy RM Genetic evidence for causal relationships between maternal obesity-related traits and birth weight. JAMA. 2016;315:1129–1140. 26978208 10.1001/jama.2016.1975 PMC4811305

[5] Jain V, Kurpad AV, Kumar B, Devi S, Sreenivas V, Paul VK Body composition of term healthy Indian newborns. Eur J Clin Nutr. 2016;70:488–493. 26373958 10.1038/ejcn.2015.152

[6] Sewell MF, Huston-Presley L, Super DM, Catalano P Increased neonatal fat mass, not lean body mass, is associated with maternal obesity. Am J Obstet Gynecol. 2006;195:1100–1103. 16875645 10.1016/j.ajog.2006.06.014

[7] Hauguel-de Mouzon S, Lepercq J, Catalano P The known and unknown of leptin in pregnancy. Am J Obstet Gynecol. 2006;194:1537–1545. 16731069 10.1016/j.ajog.2005.06.064

[8] Catalano PM, Presley L, Minium J, Hauguel-de Mouzon S Fetuses of obese mothers develop insulin resistance in utero. Diabetes Care. 2009;32:1076–1080. 19460915 10.2337/dc08-2077 PMC2681036

[9] Lawlor DA, West J, Fairley L, Nelson SM, Bhopal RS, Tuffnell D, Freeman DJ, Wright J, Whitelaw DC, Sattar N Pregnancy glycaemia and cord-blood levels of insulin and leptin in Pakistani and white British mother-offspring pairs: findings from a prospective pregnancy cohort. Diabetologia. 2014;57:2492–2500. 25273345 10.1007/s00125-014-3386-6 PMC4218974

[10] Bouret SG Nutritional programming of hypothalamic development: critical periods and windows of opportunity. Int J Obes Suppl. 2012;2:S19–S24. 27152149 10.1038/ijosup.2012.17 PMC4850605

[11] Mantzoros CS, Rifas-Shiman SL, Williams CJ, Fargnoli JL, Kelesidis T, Gillman MW Cord blood leptin and adiponectin as predictors of adiposity in children at 3 years of age: a prospective cohort study. Pediatrics. 2009;123:682–689. 19171638 10.1542/peds.2008-0343 PMC2761663

[12] Boeke CE, Mantzoros CS, Hughes MD, Rifas-Shiman LR, Villamor E, Zera CA, Gillman MW Differential associations of leptin with adiposity across early childhood. Obesity (Silver Spring). 2013;21:1430–1437. 23408391 10.1002/oby.20314 PMC3659179

[13] Nakano Y, Itabashi K, Maruyama T Association between serum adipocytokine and cholesterol levels in cord blood. Pediatr Int. 2009;51:790–794. 19419508 10.1111/j.1442-200X.2009.02853.x

[14] Lindsay RS, Nelson SM, Walker JD, Greene SA, Milne G, Sattar N, Pearson DW Programming of adiposity in offspring of mothers with type 1 diabetes at age 7. Diabetes Care. 2010;33:1080–1085. 20427684 10.2337/dc09-1766 PMC2858180

[15] Aye ILMH, Powell TL, Jansson T Review: adiponectin—the missing link between maternal adiposity, placental transport and fetal growth? Placenta. 2013;34(Suppl):S40–S45. 23245987 10.1016/j.placenta.2012.11.024 PMC3650089

[16] Qiao L, Yoo HS, Madon A, Kinney B, Hay WW, Shao J Adiponectin enhances mouse fetal fat deposition. Diabetes. 2012;61:3199–3207. 22872236 10.2337/db12-0055 PMC3501876

[17] Boyd A, Golding J, Macleod J, Lawlor DA, Fraser A, Henderson J, Molloy L, Ness A, Ring S, Davey Smith G Cohort profile: the “children of the 90s”—the index offspring of the Avon Longitudinal Study of Parents and Children. Int J Epidemiol. 2013;42:111–127. 22507743 10.1093/ije/dys064 PMC3600618

[18] Fraser A, Macdonald-Wallis C, Tilling K, Boyd A, Golding J, Davey Smith G, Henderson J, Macleod J, Molloy L, Ness A, Ring S, Nelson SM, Lawlor DA Cohort profile: the Avon Longitudinal Study of Parents and Children: ALSPAC mothers cohort. Int J Epidemiol. 2013;42:97–110. 22507742 10.1093/ije/dys066 PMC3600619

[19] Cole TJ, Bellizzi MC, Flegal KM, Dietz WH Establishing a standard definition for child overweight and obesity worldwide: international survey. BMJ. 2000;320:1240–1243. 10797032 10.1136/bmj.320.7244.1240 PMC27365

[20] Cole TJ, Freeman JV, Preece MA British 1990 growth reference centiles for weight, height, body mass index and head circumference fitted by maximum penalized likelihood. Stat Med. 1998;17:407–429. 9496720

[21] White IR, Royston P, Wood AM Multiple imputation using chained equations: issues and guidance for practice. Stat Med. 2011;30:377–399. 21225900 10.1002/sim.4067

[22] Brisbois TD, Farmer AP, McCargar LJ Early markers of adult obesity: a review. Obes Rev. 2012;13:347–367. 22171945 10.1111/j.1467-789X.2011.00965.x PMC3531624

[23] Rogers IS, Ness AR, Steer CD, Wells JCK, Emmett PM, Reilly JR, Tobias J, Smith GD Associations of size at birth and dual-energy X-ray absorptiometry measures of lean and fat mass at 9 to 10 y of age. Am J Clin Nutr. 2006;84:739–747. 17023699 10.1093/ajcn/84.4.739

[24] de Bruin NC, van Velthoven KA, Stijnen T, Juttmann RE, Degenhart HJ, Visser HK Body fat and fat-free mass in infants: new and classic anthropometric indexes and prediction equations compared with total-body electrical conductivity. Am J Clin Nutr. 1995;61:1195–1205. 7762517 10.1093/ajcn/61.6.1195

[25] Okereke NC, Uvena-Celebrezze J, Hutson-Presley L, Amini SB, Catalano PM The effect of gender and gestational diabetes mellitus on cord leptin concentration. Am J Obstet Gynecol. 2002;187:798–803. 12237665 10.1067/mob.2002.125887

[26] Sivan E, Mazaki-Tovi S, Pariente C, Efraty Y, Schiff E, Hemi R, Kanety H Adiponectin in human cord blood: relation to fetal birth weight and gender. J Clin Endocrinol Metab. 2003;88:5656–5660. 14671149 10.1210/jc.2003-031174

[27] Tsai PJ, Yu CH, Hsu SP, Lee YH, Chiou CH, Hsu YW, Ho SC, Chu CH Cord plasma concentrations of adiponectin and leptin in healthy term neonates: positive correlation with birthweight and neonatal adiposity. Horumon To Rinsho. 2004;61:88–93. 10.1111/j.1365-2265.2004.02057.x 15212649

[28] Kotani Y, Yokota I, Kitamura S, Matsuda J, Naito E, Kuroda Y Plasma adiponectin levels in newborns are higher than those in adults and positively correlated with birth weight. Horumon To Rinsho. 2004;61:418–423. 10.1111/j.1365-2265.2004.02041.x 15473872

[29] Lindsay RS, Walker JD, Havel PJ, Hamilton BA, Calder AA, Johnstone FD Adiponectin is present in cord blood but is unrelated to birth weight. Diabetes Care. 2003;26:2244–2249. 12882843 10.2337/diacare.26.8.2244

[30] Nelson SM, Freeman DJ, Sattar N, Lindsay RS Role of adiponectin in matching of fetal and placental weight in mothers with type 1 diabetes. Diabetes Care. 2008;31:1123–1125. 18339975 10.2337/dc07-2195

[31] Knittle JL, Timmers K, Ginsberg-Fellner F, Brown RE, Katz DP The growth of adipose tissue in children and adolescents. Cross-sectional and longitudinal studies of adipose cell number and size. J Clin Invest. 1979;63:239–246. 429551 10.1172/JCI109295 PMC371945

[32] Anderson EL, Howe LD, Fraser A, Callaway MP, Sattar N, Day C, Tilling K, Lawlor DA Weight trajectories through infancy and childhood and risk of non-alcoholic fatty liver disease in adolescence: the ALSPAC study. J Hepatol. 2014;61:626–632. 24768828 10.1016/j.jhep.2014.04.018 PMC4139262

[33] Flower L, Ahuja RH, Humphries SE, Mohamed-Ali V Effects of sample handling on the stability of interleukin 6, tumour necrosis factor-α and leptin. Cytokine. 2000;12:1712–1716. 11052823 10.1006/cyto.2000.0764

[34] Gislefoss RE, Grimsrud TK, Morkrid L Long-term stability of serum components in the Janus Serum Bank. Scand J Clin Lab Invest. 2008;68:402–409. 18752145 10.1080/00365510701809235

[35] Shih WJ, Bachorik PS, Haga JA, Myers GL, Stein EA Estimating the long-term effects of storage at −70 °C on cholesterol, triglyceride, and HDL-cholesterol measurements in stored sera. Clin Chem. 2000;46:351–364. 10702522

[36] Boyanton BL Jr, Blick KE Stability studies of twenty-four analytes in human plasma and serum. Clin Chem. 2002;48:2242–2247. 12446483

[37] Brinc D, Chan MK, Venner AA, Pasic MD, Colantonio D, Kyriakopolou L, Adeli K Long-term stability of biochemical markers in pediatric serum specimens stored at −80 °C: a CALIPER substudy. Clin Biochem. 2012;45:816–826. 22510430 10.1016/j.clinbiochem.2012.03.029

[38] Paltiel L, Ronningen KS, Meltzer HM, Baker SV, Hoppin JA Evaluation of Freeze Thaw Cycles on stored plasma in the Biobank of the Norwegian Mother and Child Cohort Study. Cell Preserv Technol. 2008;6:223–230. 20428472 10.1089/cpt.2008.0012 PMC2860294

[39] Gislefoss RE, Grimsrud TK, Morkrid L. Stability of selected serum proteins after long-term storage in the Janus Serum Bank. Clin Chem Lab Med. 2009;47(5):596–603. 10.1515/CCLM.2009.121 19290843

