# Supplementary material for: Programming of Adiposity in Childhood and Adolescence: Associations With Birth Weight and Cord Blood Adipokines
Source: J Clin Endocrinol Metab. 2016 Nov 14;102(2):499–506. doi: 10.1210/jc.2016-2342 (PMC5413167; doi:10.1210/jc.2016-2342)
Supplement: Supplementary file 1 [file jc.2016-2342.sf1.docx]

**Supplemental Figures and Tables**

**Supplemental Figure 1**

**Directed acyclic graph for cord leptin and adiponectin and long-term adiposity**

**
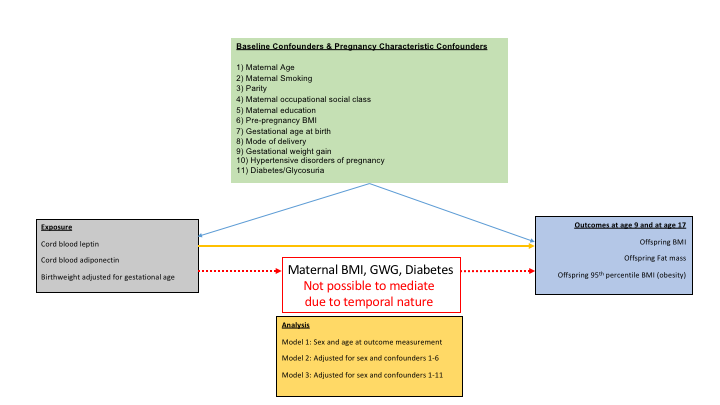
**

**Supplemental Figure 1: Explanation of directed acyclic graph**

BMI, GWG and diabetic disorders are all associated with neonatal adiposity (or main exposure in this study) and also offspring adiposity (outcome) in later life. These relationships (which have a biological basis) implicate them as confounders i.e. it is plausible that these characteristics influence neonatal adiposity (including that measured by cord-blood leptin) and later offspring adiposity and hence are, by definition, potential confounders. By contrast they could not be considered mediators as this would only be plausible if we assumed that neonatal adiposity causes maternal pre-pregnancy BMI, GWG and gestational diabetes which in turn go on to cause later offspring adiposity; not only is this not biologically plausible, it is not temporarily possible (see Supplemental Figure 1). It is possible that cord leptin (infant adiposity) mediates the effect of these more distal maternal risk factors on later offspring adiposity, however, that is a different research question than the hypothesis currently being examined.

**Supplemental Table 1:** Characteristics of observed and imputed data at age 9 and 17 years.

| **Maternal Characteristics** | Observed at age 9 | | | Multiply Imputed at Age 9 | | Observed at Age 17 | | | Multiply Imputed at Age 17 | |
| --- | --- | --- | --- | --- | --- | --- | --- | --- | --- | --- |
|  | N missing (%) | N obs (%) | Median  IQR | N obs  (%) | Median  IQR | N missing  (%) | N obs (%) | Median  IQR | N obs  (%) | Median  IQR |
| Age | 221 (8.0) | 2554 | 29  26, 32 | 2775 | 29  26, 32 | 465 (21.7) | 1673 | 29  26, 32 | 2138 | 29  26, 32 |
| Smoking  Never  Before, not during pregnancy  During pregnancy | 274 (9.9) | 1894 (75.7)  178 (7.1)  429 (17.2) |  | 2077 (74.9)  198 (7.1)  500 (18.0) |  | 497 (23.2) | 1273 (77.6)  118 (7.2)  250 (15.2) |  | 1633 (76.4)  155 (7.3)  350 (16.4) |  |
| BMI | 483 (17.4) | 2292 | 22.2  20.5, 24.4 | 2775 | 22.4  20.5, 24.7 | 630 (29.5) | 1508 | 22.0  20.5, 24.2 | 2138 | 22.2  20.5, 24.4 |
| Parity  0  1  2  3  4+ | 317 (11.4) | 1124 (45.7)  906 (36.9)  322 (13.1)  84 (3.4)  22 (0.9) |  | 1294 (46.6)  999 (36.0)  364 (13.1)  96 (3.5)  22 (0.8) |  | 522 (24.4) | 772 (47.8)  580 (35.9)  196 (12.1)  50 (3.1)  18 (1.1) |  | 1036(48.5)  749 (35.0)  265 (12.4)  64 (3.0)  24 (1.1) |  |
| Education  Left school at 16  A level  Degree | 309 (11.1) | 1479 (60.0)  628 (25.5)  359 (14.6) |  | 1,685 (60.7)  696 (25.08)  394 (14.2) |  | 519 (24.3) | 896 (55.3)  437 (30.0)  286 (17.7) |  | 1236 (57.8)  565 (26.4)  337 (15.8) |  |
| Social Class  I (least disadvantaged)  II  IIIa  IIIb  IV  V (most disadvantaged) | 646 (23.3) | 129 (6.1)  730 (34.3)  925 (43.5)  141 (6.6)  172 (8.1)  32 (1.5) |  | 148 (5.3)  916 (33.01)  1,209 (43.6)  200 (7.21)  249 (8.97)  53 (1.91) |  | 696 (32.6) | 107 (7.4)  520 (36.1)  603 (41.8)  90 (6.2)  104 (7.2)  18 (1.3) |  | 128 (6.0)  737 (34.5)  914 (42.8)  154 (7.2)  173 (8.1)  32 (1.5) |  |
| **Offspring Characteristics** |  |  |  |  |  |  |  |  |  |  |
| Sex  Male  Female | 192 (6.9) | 1243 (48.1)  1340 (51.9) |  | 1,343 (48.4)  1,432 (51.6) |  | 443 (20.7) | 722 (42.6)  973 (57.4) |  | 969 (45.3)  1169 (54.7) |  |
| **Pregnancy Characteristics** |  |  |  |  |  |  |  |  |  |  |
| Gestational age at birth (weeks) | 221 (8.0) | 2554 | 40  39, 41 | 2775 | 40  39, 41 | 465 (21.7) | 1673 | 40  39, 41 | 2138 | 40  39, 41 |
| Model of delivery  Spontaneous  Breech  Caesarean  Forceps  Vacuum  Other | 106 (3.8) | 1960 (77.4)  32 (1.3)  223 (8.8)  153 (6.0)  135 (5.3)  31 (1.2) |  | 2148 (77.4)  36 (1.3)  247 (8.9)  164 (5.9)  148 (5.3)  32 (1.2) |  | 478 (22.4) | 1303 (78.5)  20 (1.2)  129 (7.8)  90 (5.4)  96 (5.8)  22 (1.3) |  | 1657 (77.5)  28 (1.3)  175 (8.2)  120 (5.6)  129 (6.0)  29 (1.4) |  |
| Gestational weight gain (kg) | 427 (15.4) | 2348 | 12.5  9.6, 15.2 | 2775 | 12.5  9.5, 15.2 | 612 (28.6) | 1526 | 12.5  9.6, 15.3 | 2138 | 12.4  9.5, 15.3 |
| Hypertension and pre-eclampsia  No hypertensive disorders  Hypertension, no pre-eclampsia  Hypertension and pre-eclampsia | 233 (8.0) | 2159 (84.9)  342 (13.5)  41 (1.6) |  | 2351 (84.7)  378 (13.6)  46 (1.7) |  | 473 (22.1) | 1407 (84.5)  228 (13.7)  30 (1.8) |  | 1798 (84.1)  300 (14.0)  40 (1.9) |  |
| Diabetes  No glycosuria or diabetes  Existing diabetes  Gestational diabetes  Glycosuria | 433 (15.6) | 2342 (95.6)  10 (0.4)  16 (0.7)  82 (3.4) |  | 2652 (95.6)  11 (0.4)  20 (0.7)  92 (3.3) |  | 531 (24.8) | 1536 (95.6)  8 (0.5)  6 (0.4)  57 (3.6) |  | 2038 (95.3)  12 (0.6)  18 (0.8)  70 (3.3) |  |

Median, Interquartile range

Figures are numbers (%) unless stated otherwise

|  | Cord blood | | Age 9 | | | Age 17 | | |
| --- | --- | --- | --- | --- | --- | --- | --- | --- |
|  | Leptin | Adiponectin | FM | WC | BMI | FM | WC (age 15) | BMI |
| Birthweight | 1451  0.33 | 4707  0.14 | 2417  0.09 | 2527  0.16 | 2513  0.15 | 1564  0.05 | 1448  0.13 | 1614  0.10 |
| Cord blood |  |  |  |  |  |  |  |  |
| Leptin |  | 4962  0.11 | 2458  0.12 | 2572  0.05 | 2558  0.09 | 1592  0.16 | 1472  0.05 | 1645  0.08 |
| Adiponectin |  |  | 2433  0.004 | 2547  -0.04 | 2533  -0.01 | 1580  0.07 | 1456  0.02 | 1633  0.03 |
| Age 9 |  |  |  |  |  |  |  |  |
| FM |  |  |  | 2452  0.82 | 2439  0.90 | 1352  0.74 | 1271  0.60 | 1382  0.64 |
| WC |  |  |  |  | 2554  0.88 | 1420  0.52 | 1326  0.66 | 1452  0.63 |
| BMI |  |  |  |  |  | 1407  0.62 | 1313  0.63 | 1440  0.74 |
| Age 17 |  |  |  |  |  |  |  |  |
| FM |  |  |  |  |  |  | 1070  0.59 | 1589  0.78 |
| WC (age 15) |  |  |  |  |  |  |  | 1093  0.69 |

**Supplemental Table 2:** Spearman correlations between birthweight, adipokines and markers off anthropometry at age 9 and 17

**Supplemental Table 3: Cord blood analyte relative to maternal and pregnancy characteristics**

| **Maternal Characteristics** | Attended at least one clinic assessment | | |
| --- | --- | --- | --- |
|  | Cord leptin  (pg/ml)  Median (IQR) or coefficient (95%CI)  (N=2,845) | Cord Adiponectin  (µg/ml)  Median (IQR) or coefficient (95%CI)  (N=2,820) | Birthweight  (g)  Median (IQR) or  coefficient (95%CI) |
| Age (per 1 year increase) | 0.05 (-0.01, 0.11), p=0.10 | -0.26 (-0.46, -0.06), p=0.01 | 5.37 (1.87, 8.86), p=0.003 |
| BMI (per 1kg/m^2^ increase) | 0.43 (0.35, 0.52), p<0.001 | -0.04 (-0.32,0.23), p=0.74 | 19.20 (14.68, 23.72),p<0.001 |
| Smoking  Never  Before, not during pregnancy  During pregnancy | 6.33 (3.55, 12.08)  7.36 (3.42, 13.45)  6.19 (3.58, 11.23)  P=0.33 | 75.70 (53.5, 98.15)  76.98 (54.75, 98.11)  74.62 (53.53, 98.28)  P=0.22 | 3418 (3144, 3714)  3546 (3257, 3621)  3354 (3062, 3621)  P<0.001 |
| Parity  0  1  2  3  4+ | 6.11 (3.23, 11.37)  6.63 (3.74, 12.42)  6.78 (3.75, 11.91)  7.90 (4.14, 15.76)  7.01 (4.05, 12.25)  P=0.03 | 74.36, 52.91, 98.77)  77.66 (56.16, 99.41)  74.40 (51.93, 95.28)  76.24 (57.00, 98.07)  75.74 (53.29, 102.48)  P=0.45 | 3329 (3066, 3585)  3487 (3227, 3760)  3507 (3210, 3811)  3501 (3128, 3869)  3409 (3142, 3712)  P<0.001 |
| Education  Left school at 16  A level  Degree | 6.44 (3.59, 12.38)  6.31 (3.22, 11.91)  6.26 (4.00, 11.24)  P=0.67 | 76.03 (54.43, 100.45)  75.8 (52.33, 97.14)  74.18 (63.13, 95.02)  P=0.36 | 3403 (3136, 3705)  3426 (3141, 3723)  3421 (3154, 3726)  P=0.65 |
| Social Class  I (least disadvantaged)  II  IIIa  IIIb  IV  V (most disadvantaged) | 5.76 (3.92, 11.32)  6.06 (3.38, 11.79)  6.38 (3.36, 11.84)  7.58 (3.80, 13.74)  6.88 (4.12, 12.81)  7.26 (3.86, 11.34)  P=0.23 | 65.46 (49.79, 91.86)  77.56 (54.47, 98.44)  75.09 (52.91, 97.42)  76.49 (54.68, 99.55)  79.36 (55.34, 105.26)  76.65 (63.65, 100.20)  P=0.23 | 3448 (3144, 3786)  3401 (3142, 3686)  3405 (3152, 3713)  3397 (3128, 3692)  3491 (3126, 3769)  3422 (3141, 3766)  P=0.65 |
| **Pregnancy Characteristics** |  |  |  |
| Model of delivery  Spontaneous  Breech  Caesarean  Forceps  Vacuum  Other | 6.41 (3.58, 12.02)  6.72 (3.42, 11.56)  5.84 (3.36, 11.21)  5.85 (3.18, 12.37)  7.72 (4.14, 15.31)  5.70 (2.98, 11.89)  P=0.09 | 76.95 (54.64, 99.51)  66.24 (41.42, 94.81)  67.21 (47.38, 89.83)  78.83 (58.27, 102.56)  72.91 (46.71, 94.78)  74.94 (51.66, 101.88)  P<0.001 | 3415 (3144, 3714)  3230 (3042, 3437)  3425 (3088, 3741)  3409 (3153, 3705)  3335 (3076, 3598)  3271 (3191, 3634)  P=0.006 |
| Gestational weight gain (per 1kg increase) | 0.22 (0.16, 0.29), p<0.001 | 0.33 (0.11, 0.54), p=0.003 | 21.19 (18.40, 23.98), p<0.001 |
| Hypertension and pre-eclampsia  No hypertensive disorders  Hypertension, no pre-eclampsia  Hypertension and pre-eclampsia | 6.32 (3.58, 12.03)  6.84 (3.49, 12.82)  6.87 (3.15, 14.22)  P=0.61 | 76.43 (54.19, 98.38)  73.26 (52.75, 97.22)  65.58 (50.68, 95.24)  P=0.17 | 3408 (3142, 3708)  3425 (3134, 3716)  3288 (2952, 3775)  P=0.61 |
| Diabetes  No glycosuria or diabetes  Existing diabetes  Gestational diabetes  Glycosuria | 6.26 (3.56, 11.98)  8.90 (7.2, 20.22)  8.62 (3.15, 21.94)  7.88 (3.90, 13.35)  P=0.08 | 75.99 (53.82, 98.88)  51.15 (36.07, 94.52)  71.86 (48.86, 86.34)  71.42 (52.24, 93.34)  P=0.33 | 3405 (3138, 3705)  3541 (3094, 4122)  3746 (3541, 4347)  3464 (3181, 3733)  P<0.001 |
| Median (Interquartile range)  P values for differences between categories  Birthweight adjusted for sex, gestational age and number of offspring | | |  |

**Supplemental Table 4:** Associations of birthweight and cord blood analyte with fat mass, waist circumference and BMI at age 9 years. N= 2775

| Outcome | Fat mass* | | | Waist circumference | | | BMI | | |
| --- | --- | --- | --- | --- | --- | --- | --- | --- | --- |
| Exposure | %  Change | 95% CI | P | %  Change | 95% CI | P | %  Change | 95% CI | P |
| Leptin (10pg/ml)  Model 1  Model 2  Model 3 | 3.9  2.0  1.5 | 2.2, 5.7  0.3, 3.7  -0.2, 3.3 | <0.001  0.022  0.077 | 1.0  0.5  0.4 | 0.6, 1.4  0.1, 0.9  0.0, 0.8 | <0.001  0.016  0.069 | 1.5  0.8  0.6 | 0.9, 2.0  0.3, 1.3  0.1, 1.1 | <0.001  0.003  0.023 |
| Adiponectin (10µg/ml)  Model 1  Model 2  Model 3 | 0.0  0.1  0.1 | -0.5, 0.5  -0.4, 0.6  -0.4, 0.6 | 0.999  0.738  0.758 | -0.1  -0.1  -0.1 | -0.2, 0.0  -0.2, 0.0  -0.2, 0.0 | 0.152  0.236  0.186 | 0.0  0.0  0.0 | -0.2, 0.1  -0.1, 0.2  -0.2, 0.2 | 0.830  0.860  0.975 |
| Birthweight‡ (100g)  Model 1  Model 2  Model 3 | 0.5  0.1  -0.1 | 0.0, 0.9  -0.3, 0.5  -0.5, 0.4 | 0.032  0.609  0.710 | 0.4  0.3  0.3 | 0.3, 0.5  0.2, 0.4  0.2, 0.4 | <0.001  <0.001  <0.001 | 0.5  0.4  0.4 | 0.4, 0.7  0.3, 0.5  0.2, 0.5 | <0.001  <0.001  <0.001 |

Model 1: Adjusted for offspring sex and age at measurement.

Model 2: Adjusted for offspring sex, age at measurement and maternal confounders (age, smoking, parity, occupational social class, education and pre-pregnancy BMI).

Model 3: Adjusted for offspring sex, age at measurement and maternal confounders plus pregnancy confounders (gestational age at birth, mode of delivery, gestational weight gain, hypertensive disorders and diabetic disorders of pregnancy).

* Fat mass adjusted for height

‡ Birthweight adjusted for sex, gestational age and singleton/twin pregnancy

% refers to percentage change of outcome per unit increase in exposure

**Supplemental Table 5:** Associations of birthweight and cord blood analyte with fat mass, waist circumference (at age 15 years) and BMI at age 17 years. N= 2138

| Outcome | Fat mass* | | | Waist Circumference | | | BMI | | |
| --- | --- | --- | --- | --- | --- | --- | --- | --- | --- |
| Exposure | %  Change | 95% CI | P | %  Change | 95% CI | P | %  Change | 95% CI | P |
| Leptin (10pg/ml)  Model 1  Model 2  Model 3 | 3.6  1.0  0.6 | 1.5, 5.8  -1.0, 3.1  -1.5, 2.7 | 0.001  0.335  0.568 | 0.9  0.4  0.4 | 0.5, 1.4  0.0, 0.9  -0.1, 0.9 | <0.001  0.074  0.100 | 1.4  0.5  0.3 | 0.8, 2.1  -0.2, 1.1  -0.3, 1.0 | <0.001  0.151  0.317 |
| Adiponectin (10µg/ml)  Model 1  Model 2  Model 3 | 0.6  0.7  0.6 | -0.1, 1.2  0.0, 1.3  0.0, 1.3 | 0.092  0.039  0.048 | 0.1  0.2  0.2 | 0.0, 0.3  0.0, 0.3  0.0, 0.3 | 0.061  0.024  0.032 | 0.2  0.2  0.2 | 0.0, 0.4  0.0, 0.4  0.0, 0.4 | 0.083  0.029  0.033 |
| Birthweight‡ (100g)  Model 1  Model 2  Model 3 | 1.2  0.6  0.5 | 0.7, 1.7  0.1, 1.2  -0.1, 1.0 | <0.001  0.016  0.091 | 0.5  0.4  0.4 | 0.4, 0.6  0.3, 0.5  0.3, 0.5 | <0.001  <0.001  <0.001 | 0.5  0.3  0.3 | 0.3, 0.7  0.1, 0.4  0.1, 0.4 | <0.001  <0.001  0.002 |

Model 1: Adjusted for offspring sex and age at measurement.

Model 2: Adjusted for offspring sex, age at measurement and maternal confounders (age, smoking, parity, occupational social class, education and pre-pregnancy BMI).

Model 3: Adjusted for offspring sex, age at measurement and maternal confounders plus pregnancy confounders (gestational age at birth, mode of delivery, gestational weight gain, hypertensive disorders and diabetic disorders of pregnancy).

* Fat mass adjusted for height

‡ Birthweight adjusted for sex, gestational age and singleton/twin pregnancy

% refers to percentage change of outcome per unit increase in exposure

**Supplemental Table 6:** Associations of birthweight and cord blood analyte with fat mass, waist circumference and BMI z-scores and obesity at age 9 years using complete case analysis.

| Outcome | Fat mass z-score * | | | Waist circumference z-score | | | BMI z-score | | | Obesity | | |
| --- | --- | --- | --- | --- | --- | --- | --- | --- | --- | --- | --- | --- |
| Exposure | Coefficient | 95% CI | P | Coefficient | 95% CI | P | Coefficient | 95% CI | P | OR | 95% CI | P |
| Leptin (10pg/ml)  Model 1  Model 2  Model 3 | 0.06  0.03  0.02 | 0.03, 0.10  -0.01, 0.07  -0.01, 0.06  N=1,684 | <0.0010.096  0.239 | 0.09  0.05  0.04 | 0.05, 0.13  0.01, 0.09  0.00, 0.08  N=1,776 | <0.001  0.014  0.051 | 0.09  0.05  0.03 | 0.05, 0.14  0.01, 0.09  -0.01, 0.08  N=1,764 | <0.0010.029  0.141 | 1.09  0.93  0.89 | 0.91, 1.32  0.75, 1.14  0.72, 1.10  N=1,764 | 0.343  0.463  0.289 |
| Adiponectin (10µg/ml)  Model 1  Model 2  Model 3 | 0.00  0.00  0.00 | -0.01, 0.01  -0.01, 0.02  -0.01, 0.02  N=1,666 | 0.910  0.573  0.498 | -0.01  0.00  0.00 | -0.02, 0.01  -0.01, 0.01  -0.02, 0.01  N=1,758 | 0.393  0.761  0.728 | 0.00  0.01  0.01 | -0.01, 0.02  -0.01, 0.02  -0.01, 0.02  N=1,746 | 0.786  0.346  0.356 | 1.04  1.06  1.06 | 0.97, 1.12  0.99, 1.14  0.98, 1.14  N=1,746 | 0.267  0.116  0.134 |
| Birthweight‡ (100g)  Model 1  Model 2  Model 3 | 0.01  0.00  0.00 | 0.00, 0.02  -0.01, 0.01  -0.01, 0.01  N=1,673 | 0.100  0.707  0.679 | 0.04  0.03  0.03 | 0.02, 0.05  0.02, 0.04  0.01, 0.04  N=1,765 | <0.001  <0.001  <0.001 | 0.04  0.03  0.03 | 0.03, 0.05  0.02, 0.05  0.02, 0.04  N=1,753 | <0.001<0.001  <0.001 | 1.05  1.02  1.00 | 0.99, 1.11  0.96, 1.08  0.94, 1.06 N=1,753 | 0.128  0.553  0.952 |

Model 1: Adjusted for offspring sex and age at measurement.

Model 2: Adjusted for offspring sex, age at measurement and maternal confounders (age, smoking, parity, occupational social class, education and pre-pregnancy BMI).

Model 3: Adjusted for offspring sex, age at measurement and maternal confounders plus pregnancy confounders (gestational age at birth, mode of delivery, gestational weight gain, hypertensive disorders and diabetic disorders of pregnancy).

* Fat mass adjusted for height

‡ Birthweight adjusted for sex, gestational age and singleton/twin pregnancy

**Supplemental Table 7:** Associations of birthweight and cord blood analyte with fat mass, waist circumference (at age 15 years) and BMI z-scores and obesity at age 17 years using complete case analysis.

| Outcome | Fat mass z-score * | | | Waist circumference z-score | | | BMI z-score | | | Obesity | | |
| --- | --- | --- | --- | --- | --- | --- | --- | --- | --- | --- | --- | --- |
| Exposure | Coefficient | 95% CI | P | Coefficient | 95% CI | P | Coefficient | 95% CI | P | OR | 95% CI | P |
| Leptin (10pg/ml)  Model 1  Model 2  Model 3 | 0.08  0.03  0.02 | 0.03, 0.13  -0.02, 0.08  -0.03, 0.07  N=1,143 | 0.001  0.224  0.393 | 0.06  0.03  0.04 | 0.01, 0.11  -0.02, 0.08  -0.02, 0.09  N=1,055 | 0.023  0.271  0.171 | 0.09  0.02  0.01 | 0.03, 0.14  -0.04, 0.07  -0.05, 0.06  N=1,177 | 0.003  0.485  0.765 | 1.17  0.96  0.93 | 1.00, 1.38  0.78, 1.18  0.75, 1.16  N=1,177 | 0.049  0.679  0.543 |
| Adiponectin (10µg/ml)  Model 1  Model 2  Model 3 | 0.01  0.01  0.01 | -0.01, 0.03  -0.01, 0.03  -0.01, 0.03  N=1,132 | 0.257  0.244  0.208 | 0.01  0.01  0.01 | -0.01, 0.03  0.00, 0.03  0.00, 0.03  N=1,041 | 0.266  0.164  0.135 | 0.01  0.01  0.01 | -0.01, 0.03  -0.01, 0.03  -0.01, 0.03  N=1,166 | 0.497  0.439  0.383 | 1.02  1.03  1.04 | 0.95, 1.10  0.95, 1.11  0.96, 1.12  N=1,166 | 0.554  0.482  0.386 |
| Birthweight‡ (100g)  Model 1  Model 2  Model 3 | 0.03  0.01  0.01 | 0.01, 0.04  0.00, 0.03  0.00, 0.02  N=1,136 | <0.0010.040  0.149 | 0.03  0.02  0.02 | 0.01, 0.04  0.01, 0.04  0.01, 0.04  N=1,048 | <0.001  0.001  <0.001 | 0.03  0.02  0.02 | 0.02, 0.05  0.01, 0.04  0.00, 0.03  N=1,170 | <0.0010.004  0.022 | 1.09  1.05  1.05 | 1.04, 1.15  0.99, 1.12  0.98, 1.11  N=1,170 | 0.001  0.093  0.159 |

Model 1: Adjusted for offspring sex and age at measurement.

Model 2: Adjusted for offspring sex, age at measurement and maternal confounders (age, smoking, parity, occupational social class, education and pre-pregnancy BMI).

Model 3: Adjusted for offspring sex, age at measurement and maternal confounders plus pregnancy confounders (gestational age at birth, mode of delivery, gestational weight gain, hypertensive disorders and diabetic disorders of pregnancy).

* Fat mass adjusted for height

‡ Birthweight adjusted for sex, gestational age and singleton/twin pregnancy

**Supplemental Table 8:** Associations of birthweight and cord blood analyte with fat mass, waist circumference and BMI at age 9 years using complete case analysis.

| Outcome | Fat mass* | | | Waist circumference | | | BMI | | |
| --- | --- | --- | --- | --- | --- | --- | --- | --- | --- |
| Exposure | %  Change | 95% CI | P | %  Change | 95% CI | P | %  Change | 95% CI | P |
| Leptin (10pg/ml)  Model 1  Model 2  Model 3 | 3.3  1.4  0.9 | 1.2, 5.4  -0.6, 3.4  -1.1, 2.9  N=1,684 | 0.002  0.160  0.373 | 1.0  0.6  0.4 | 0.5, 1.5  0.1, 1.0  0.0, 0.9  N=1,776 | <0.001  0.019  0.068 | 1.0  0.5  0.3 | 0.7, 1.9  0.1, 1.3  -0.1, 1.1  N=1,764 | <0.001  0.024  0.121 |
| Adiponectin (10µg/ml)  Model 1  Model 2  Model 3 | 0.1  0.3  0.4 | -0.6, 0.8  -0.3, 1.0  -0.3, 1.0  N=1,666 | 0.752  0.336  0.280 | 0.0  0.0  0.0 | -0.2, 0.1  -0.2, 0.1  -0.2, 0.1  N=1,758 | 0.559  0.937  0.922 | 0.0  0.1  0.1 | -0.2, 0.3  -0.1, 0.3  -0.1, 0.3  N=1,746 | 0.651  0.270  0.272 |
| Birthweight‡ (100g)  Model 1  Model 2  Model 3 | 0.4  0.0  -0.2 | -0.1, 0.9  -0.5, 0.6  -0.8, 0.3  N=1,673 | 0.153  0.965  0.433 | 0.4  0.4  0.3 | 0.3, 0.6  0.2, 0.5  0.2, 0.4  N=1,765 | <0.001  <0.001  <0.001 | 0.5  0.4  0.4 | 0.4, 0.7  0.3, 0.6  0.2, 0.5  N=1,753 | <0.001  <0.001  <0.001 |

Model 1: Adjusted for offspring sex and age at measurement.

Model 2: Adjusted for offspring sex, age at measurement and maternal confounders (age, smoking, parity, occupational social class, education and pre-pregnancy BMI).

Model 3: Adjusted for offspring sex, age at measurement and maternal confounders plus pregnancy confounders (gestational age at birth, mode of delivery, gestational weight gain, hypertensive disorders and diabetic disorders of pregnancy).

* Fat mass adjusted for height

‡ Birthweight adjusted for sex, gestational age and singleton/twin pregnancy

% refers to percentage change of outcome per unit increase in exposure

**Supplemental Table 9:** Associations of birthweight and cord blood analyte with fat mass, waist circumference (at age 15 years) and BMI at age 17 years using complete case analysis.

| Outcome | Fat mass* | | | Waist circumference | | | BMI | | |
| --- | --- | --- | --- | --- | --- | --- | --- | --- | --- |
| Exposure | %  Change | 95% CI | P | %  Change | 95% CI | P | %  Change | 95% CI | P |
| Leptin (10pg/ml)  Model 1  Model 2  Model 3 | 3.9  1.3  0.8 | 1.4, 6.6  -1.2, 3.8  -1.7, 3.4  N=1,143 | 0.002  0.317  0.536 | 0.7  0.3  0.4 | 0.1, 1.3  -0.3, 0.9  -0.3, 1.0  N=1,055 | 0.022  0.309  0.194 | 1.4  0.5  0.4 | 0.7, 2.1  -0.2, 1.1  -0.3, 1.1  N=1,297 | <0.001  0.173  0.244 |
| Adiponectin (10µg/ml)  Model 1  Model 2  Model 3 | 0.4  0.4  0.4 | -0.5, 1.3  -0.5, 1.2  -0.4, 1.3  N=1,132 | 0.397  0.376  0.343 | 0.1  0.1  0.1 | -0.1, 0.3  -0.1, 0.3  -0.1, 0.3  N=1,041 | 0.357  0.231  0.192 | 0.0  0.1  0.1 | -0.2, 0.3  -0.1, 0.3  -0.1, 0.3  N=1,281 | 0.750  0.410  0.410 |
| Birthweight‡ (100g)  Model 1  Model 2  Model 3 | 1.3  0.7  0.5 | 0.6, 2.0  0.0, 1.4  -0.2, 1.3  N=1,136 | <0.001  0.049  0.137 | 0.3  0.2  0.3 | 0.2, 0.5  0.1, 0.4  0.1, 0.4  N=1,048 | <0.001  0.003  0.001 | 0.5  0.3  0.3 | 0.3, 0.7  0.1, 0.5  0.1, 0.5  1,289 | <0.001  0.001  0.003 |

Model 1: Adjusted for offspring sex and age at measurement.

Model 2: Adjusted for offspring sex, age at measurement and maternal confounders (age, smoking, parity, occupational social class, education and pre-pregnancy BMI).

Model 3: Adjusted for offspring sex, age at measurement and maternal confounders plus pregnancy confounders (gestational age at birth, mode of delivery, gestational weight gain, hypertensive disorders and diabetic disorders of pregnancy).

* Fat mass adjusted for height

‡ Birthweight adjusted for sex, gestational age and singleton/twin pregnancy

% refers to percentage change of outcome per unit increase in exposure
